# SIAH1 ubiquitination-modified HMGCR inhibits lung cancer progression and promotes drug sensitivity through cholesterol synthesis

**DOI:** 10.1186/s12935-023-02914-w

**Published:** 2023-04-16

**Authors:** Hongmei Yuan, Hongge Wu, Jing Cheng, Jie Xiong

**Affiliations:** 1 Department of Pathology, Wuhan Jinyintan Hospital, Tongji Medical College of Huazhong University of Science and Technology; Hubei Clinical Research Center for Infectious Diseases; Wuhan Research Center for Communicable Disease Diagnosis and Treatment, Chinese Academy of Medical Sciences; Joint Laboratory of Infectious Diseases and Health, Wuhan Institute of Virology and Wuhan Jinyintan Hospital, Chinese Academy of Sciences, Wuhan, 430023 Hubei Province China; 2 Cancer Center, Union Hospital, Tongji Medical College, Huazhong University of Science and Technology, No. 1277 Jiefang Avenue, Wuhan, 430022 Hubei province China

**Keywords:** HMGCR, SIAH1, Cholesterol, Lung cancer, Cisplatin

## Abstract

**Backgrounds:**

Lung cancer is one of the most frequently diagnosed cancers and the leading cause of cancer-related deaths worldwide. Deep understanding of chemoresistance will lead to remarkable progress in lung cancer treatment strategy. Cholesterol accumulation was associated with cisplatin resistance in lung cancer treatment. And we found the degree of cisplatin resistance was correlated with the expression of the cholesterol synthesis HMGCR.

**Methods:**

We analyzed a group of 42 lung cancer patients who received cisplatin treatment after lung resection surgery. The expression of HMGCR and its correlation with cholesterol in lung cancer cell lines were determined by qRT-PCR and ELISA analyses. We focus on the function and mechanism of HMGCR in lung cancer and reveal that knockdown of HMGCR expression inhibits the proliferation, colony formation, and migration of lung cancer cell lines in vitro or in vivo and dramatically enhances the efficacy of cisplatin.

**Results:**

Through mechanism studies, we illustrate that SIAH1, an E3 ubiquitin-protein ligase, ubiquitination modifies HMGCR and inhibits efflux protein activity via regulating cholesterol synthesis. In vivo experiments showed that SIAH1 overexpression or using HMGCR knockdown retard tumor growth and enhanced the efficacy of cisplatin. In summary, HMGCR affects cholesterol metabolism by regulating key enzymes in cholesterol synthesis, thereby reducing drug sensitivity.

**Conclusion:**

This study indicates that lung cancer patients with lower HMGCR levels may lead to a better prognosis and provide a potential treatment by SIAH1 overexpression for lung cancer patients with cisplatin resistance.

**Supplementary Information:**

The online version contains supplementary material available at 10.1186/s12935-023-02914-w.

## Introduction

Lung cancer is one of the most frequently diagnosed cancers and the leading cause of cancer-related deaths [1]. In the past 50 years, lung cancer has significantly increased in incidence and mortality [2]. Although molecular targeted therapies and immunotherapies for non-small-cell lung cancer have improved the 5 years survival rate, resistance to current treatments frequently occurs [3]. Understandingthe ease of biology will lead to remarkable progress in lung cancer treatment strategy.

Platinum drugs are often used as first-line anticancer drugs in lung adenocarcinoma. However, drug resistance greatly limits their clinical application, so it is important to identify resistance mechanisms to expand clinical treatment options. Recent studies have found that the accumulation of cholesterol is regarded as an important factor of tumor resistance, and cancer cells always have higher cholesterol [4], including non-small cell lung cancer, gallbladder cancer, hepatocellular carcinoma, and breast cancer. Cholesterol homeostasis is tightly controlled by several physiological processes, including its input, synthesis, output, metabolism, and esterification. And the increase of cholesterol content in tumor cells is closely related to its biosynthesis, increased uptake, and decreased efflux, and further affected tumor progression. It has been shown that statins are associated with reduced mortality from liver cancer after adjustment of cholesterol [5]. Furthermore, cholesterol affects the functional outcome of anticancer drugs in various cancer cell [6]. However, the relationship between high cholesterol and cisplatin resistance in lung cancer remains unresolved.

Hydroxy-3-methylglutaryl coenzyme A reductase (HMGCR), a rate-limiting enzyme for endogenous cholesterol synthesis, is essential in cholesterol [7] and also has the effect of promoting [8, 9]. HMGCR, in previous studies, was reported to be upregulated in a variety of tumors, such as gastric [10], bladder [11], and liver [12], thus promoting the malignant phenotype of cancer cells. As cholesterol role in cancer, inhibition of cholesterol synthesis might be a therapeutic strategy to treat cancers, such as statins, one of the HMGCR inhibitors. However, it has not been reported clearly in lung cancer. Besides pro-tumor roles, HMGCR also influences drug resistance by regulating the activity of p-glycoprotein (P-gp, encoded by ABCB1). Some studies suggested that HMGCR inhibitor atorvastatin treatment decreases p-glycoprotein [13, 14]. ABCB1 and ABCG2 are members of the ATP-binding cassette drug transport protein family, which reduce drug accumulation by using energy generated from the hydrolysis of ATP to confer resistance against chemotherapy [15]. Therefore, developing a novel P-gp inhibitor or antitumor drug that is not a P-gp substrate to avoid chemotherapy drug efflux and increase drug sensitivity is of great significance for tumor-resistant patients.

This study focuses on the function and mechanism of HMGCR in lung cancer. We found that HMGCR knockdown inhibited the proliferation and migration of lung cancer cell lines in vitro and in vivo, and was associated with cisplatin resistance. Mechanism studies showed SIAH1 degrades HMGCR through ubiquitination, and decrease cholesterol synthesis. Through lowing cholesterol, HMGCR reduced the expression of the P-gp protein in cisplatin treatment, thereby enhancing cisplatin sensitivity. Our findings suggested an essential role for the SIAH1/HMGCR axis in lung cancer chemoresistance.

## Materials and methods

### Patient samples

Lung cancer tissues were obtained from 42 non-progressive and progressive lung cancer patients with cisplatin treatment in Wuhan Jinyintan Hospital from 2016 to 2022. The Ethics Committee of Wuhan Jinyintan hospital(Approval no. KY-2021-04) approved the protocols, and written informed consents were acquired. The collected samples were fresh-frozen or paraffin-embedded for further analysis.

### Cell culture

The lung cancer cell lines H1975, A549, and A549/DPP were purchased from Shanghai Cell Bank, Chinese Academy of Sciences (Shanghai, China) and cultured in RPMI 1640 Medium (Invitrogen) supplemented with 10% fetal bovine serum (Gibco), 1% Glutamax (Invitrogen), 1% Sodium Pyruvate 100 mM Solution and 1% penicillin/streptomycin (Gibco). H293T, DEAS-2B, H441, and H1373 were purchased from Nanjing Cobioer Biosciences (Nanjing, China). All cell lines were maintained at 37 °C with 5% CO_2_.

### Plasmid construction and transfection

The gene of HMGCR was cloned into pCDH-CMV-MCS-EF1-Puro. shRNA targeting SIAH1, HMGCR, and negative control sh-NC were synthesized by Generay (Shanghai, China). The shRNAs targeting sequences used for these experiments are shown in Table S1. The transfection was conducted by Lipofectamine 3000 (Invitrogen, USA).

### Stable cell line construction

A549, A549/DDP, or 293T cells was transfected with lentivirus containing HMGCR or shRNA targeting HMGCR, SIAH1 in the presence of 8ug/mL polybrene. After 3 days, the transfected cells were selected with 2ug/mL puromycin for another 3 days, and the mRNA and protein levels were detected to confirm the expression of the desired protein.

### CRISPR Cas9

Cells were transformed with CRISPR/Cas9 and HDR plasmids targeting HMGCR (Santa Cruz Biotechnology Inc., CA, USA). Cells were cotransfected with Cas9 nuclease and HDR plasmids, each encoding a guide RNA targeting exon 1, exon 2, or the exon 2/intron 2 junction. two days after transfection, cells were chosen with 4 µg/ml puromycin (Merck/Millipore, Stockholm, Sweden), and single-cell colonies were expanded. Knockout levels were verified using WB and qRT-PCR.

### COIP and ubiquitination assay

Cell lysates were obtained through RIPA lysis buffer, incubated with anti-SIAH1 (Abcam, ab69638), anti-HMGCR (Abcam, ab242315), or normal IgG (Santa Cruz Biotechnology, sc-2027) antibody, followed by incubation with protein A/G PLUS-Agarose beads (Thermo, 20,421) for 4 h at 4 °C. The immunocomplexes reacted with anti-SIAH1, anti-HMGCR, or anti-ubiquitin (Santa Cruz, F2819) antibodies.

For the in vitro ubiquitination assay, SIAH1, and HMGCR were individually and separately expressed and purified from 293T cells. The ubiquitination reaction buffer was for 1 h at 30 °C, stopped with 50 µL of 2 × SDS-PAGE buffer, and boiled at 95 °C for 10 min. The immunocomplexes were immediately separated by SDS-PAGE and blotted with the indicated antibodies.

### Immunofluorescence

Cells were grown on glass coverslips and transfected with the indicated siRNA. Transfected and fixed with 4% paraformaldehyde at room temperature for 30 min, blocked in a 4% BSA solution in PBS for 1 h, and incubated with anti-SIAH1 and anti-HMGCR antibody at 4 °C overnight. Then incubated with Alexa Fluor 488-labeled goat anti-mouse IgG antibody at room temperature for 1 h, stained with 1 µg/ml DAPI for 5 min, washed with PBS, and dried. The cells were detected at 100× magnification using a Zeiss Axio Imager A1 fluorescence microscope.

### Western blot

Whole-cell lysates were prepared with RIPA Lysis Buffer (Beyotime, P0013B, China), then the proteins were separated using 10% SDS-polyacrylamide gels, and to 0.45 μm PVDF membranes, then blocked and incubated with the following primary antibodies: Anti-SIAH1 (Abcam, ab69638, 1:200), anti-GAPDH (Cell Signaling, D16H11, 1:1000). Anti-ABCB1 (sc-55,510, 1:1000), anti-ABCB4 (sc-58,221, 1:1000), anti-HMGCR (sc-271,595, 1:1000) were purchased from Santa Cruz Biotechnology; anti-ABCG1 (13578-1-AP, 1:1000), anti-ABCG2 (27286-1-AP, 1/1000 dilution) were from Proteintech Group. Then membranes were incubated with secondary antibodies (Cell Signaling Technology) and incubated with the ECL western blotting substrate. Chemiluminescent images were acquired with the iBright CL1000.

### qRT-PCR

Total RNAs from fresh-frozen tissues or cells were extracted and reversely transcribed into cDNA using PrimeScript RT Reagent Kit (Takara Bio, China). qRT-PCR was performed on the 7500 Fast Real-time PCR System (Applied Biosystems, USA). The 2^−ΔΔCT^ method was used for expression analysis. The primers used for these experiments are shown in Table S1.

### ELISA

The concentration of TC and TG were measured using the appropriate ELISA kits. ELISAs were conducted according to the instruction book.

### Cell proliferation assay

For the CCK-8 assay, two thousand A549 cells or A549/DDP cells per well were cultured for 24 h, 48 h, and 72 h in 96-well plates. Cell Counting Kit-8 solution (Beyotime, C0038, China) was added and cultured for 4 h, then the absorbance at 450 nm was determined through a microplate reader. Five hundred A549 or A549/DDP cells were cultured for 10 days in 6-well plates for colony forming process assay. Crystal violet was used to stain the colonies and dissolved with 10% acetic acid. The absorbance at 590 m was determined through a microplate reader.

### IC50 determination

Two thousand cells per well were seeded in 96- well plates for 24 h and then treated with compounds (including cisplatin, pseudoprotodioscin, and cholesterol). Cell number was measured using CCK-8 following the manufacturers’ protocol. IC50 determinations were performed using GraphPad Prism software.

### Clone formation

1000 cells per well were seeded into 6-well plates for 2 weeks, and the medium was replaced every 3 days. Next, cells were fixed with 4% formaldehyde for 15 min and stained with 0.1% crystal violet. The clonogenicity was gained using a microscope at 100×magnification.

### Transwell invasion assay

Transwell chambers were placed in 24-well plates and coated with basement membrane Matrigel (diluted to 2 mg/ml). Seed 1 × 10^5^ A549 or A549/DDP cells into the upper chamber with 200 µl of serum-free RPMI 1640 Medium. Add 1000 µl of RPMI 1640 Medium containing 10% FBS to the lower section. Then the plate was incubated at 37 °C for 24 h. At last, the cells were fixed, stained, imaged, and counted.

### IHC

After deparaffinization and rehydration, immerse slides in citric acid unmasking solution at 95 °C for 15 min. After natural cooling, immerse the slides in 3% hydrogen peroxide for 10 min to quench endogenous peroxidase activity. Block the slides with 5% BSA for 1 h and incubate with primary antibody overnight at 4 °C. Then, wash the slides with PBS and incubate them with secondary antibody for 30 min at 25 °C. Incubate the slides with a diaminobenzidine solution, and last, counterstain the slides with hematoxylin.

### Tumor xenografts assay

Female nude mice (6 weeks old) were grouped (n = 6/group). Nude mice were subcutaneously injected with 5 × 10^5^ cells stable, overexpressing or knocking down HMGCR and SIAH1. Tumor volumes were determined 7, 14, 21, and 28 days after the first injection and were calculated as length×width^2^ × 0.5. Cisplatin was administered via intraperitoneal injections of 2 mg per three days. Tumor lengths and body weights were recorded every 2 days. On day 28, blood samples were collected from the eye orbits under ether anesthesia, and then the mice were immediately euthanized by the cervical dislocation method. Tumor tissues were collected for subsequent experiments. These experiments were approved by Ethics Committee of Wuhan Jinyintan hospital (Approval no. KY-2021-04) and by the National Institutes of Health Guidelines for the Care and Use of Laboratory Animals.

### Statistical analysis

The experiment was repeated three times. Results are shown as mean ± standard deviation and analyzed through Student’s t-test to compare two groups or two-way ANOVA among multiple groups by GraphPad Prism software. P-values < 0.05 were considered significant.

## Results

### Clinical study

We analyzed a group of 42 patients with lung cancer who received cisplatin treatment after resection surgery from 2016 to 2022 (Table 1). The patients were disordered into two groups based on whether the disease developed after cisplatin treatment. Cisplatin resistance was not significantly correlated with other clinicopathological characteristics such as age (*P* = 0.503), smoking status (*P* = 0.647), and body mass index (BMI) (*P* = 0.788). We further carefully evaluated total cholesterol (TC), triglyceride (TG), and high-density lipoprotein (HDL) levels in the progressed patients (n = 27) versus non-progressive patients (n = 15) (Table 1). Plasma TC, rather than TGs and HDL, was higher in the progression group, suggesting that elevated plasma TC may be a crucial risk factor in cisplatin-resistant lung cancer.

**Table 1:** Clinical characteristics and blood lipid level in non-progressive and progressive lung cancer patients with cisplatin treatment

	*Non-progression (n = 15)*	*Progression (n = 27)*	*P*
*Age (years)*	*53.94 ± 6.15*	*55.48 ± 7.47*	*0.503*
*Smoking rate (%)*	*60 (%)*	*52 (%)*	*0.647*
*BMI (kg/m* ^*2*^ *)*	*22.43 ± 2.51*	*22.23 ± 2.19*	*0.788*
*TG (mmol/L)*	*1.16 ± 0.36*	*1.24 ± 0.29*	*0.398*
*TC (mmol/L)*	*4.21 ± 0.54*	*5.02 ± 0.83*	*0.002* ^*^
*HDL (mmol/L)*	*1.29 ± 0.27*	*1.36 ± 0.26*	*0.476*
*LDL (mmol/L)*	*2.41 ± 0.54*	*2.33 ± 0.62*	*0.706*

*BMI* body mass index, *TC* Total cholesterol, *TG* Total triglyceride, *HDL* High-density lipoprotein cholesterol, *LDL* Low-density lipoprotein cholesterol. Non-progression group: there is no progress after a period of treatment of cisplatin, progression group: there is medical progress after a period of treatment of cisplatin

### Accumulation of cholesterol reduces sensitivity to cisplatin in lung cancer cell lines

**Fig. 1:**
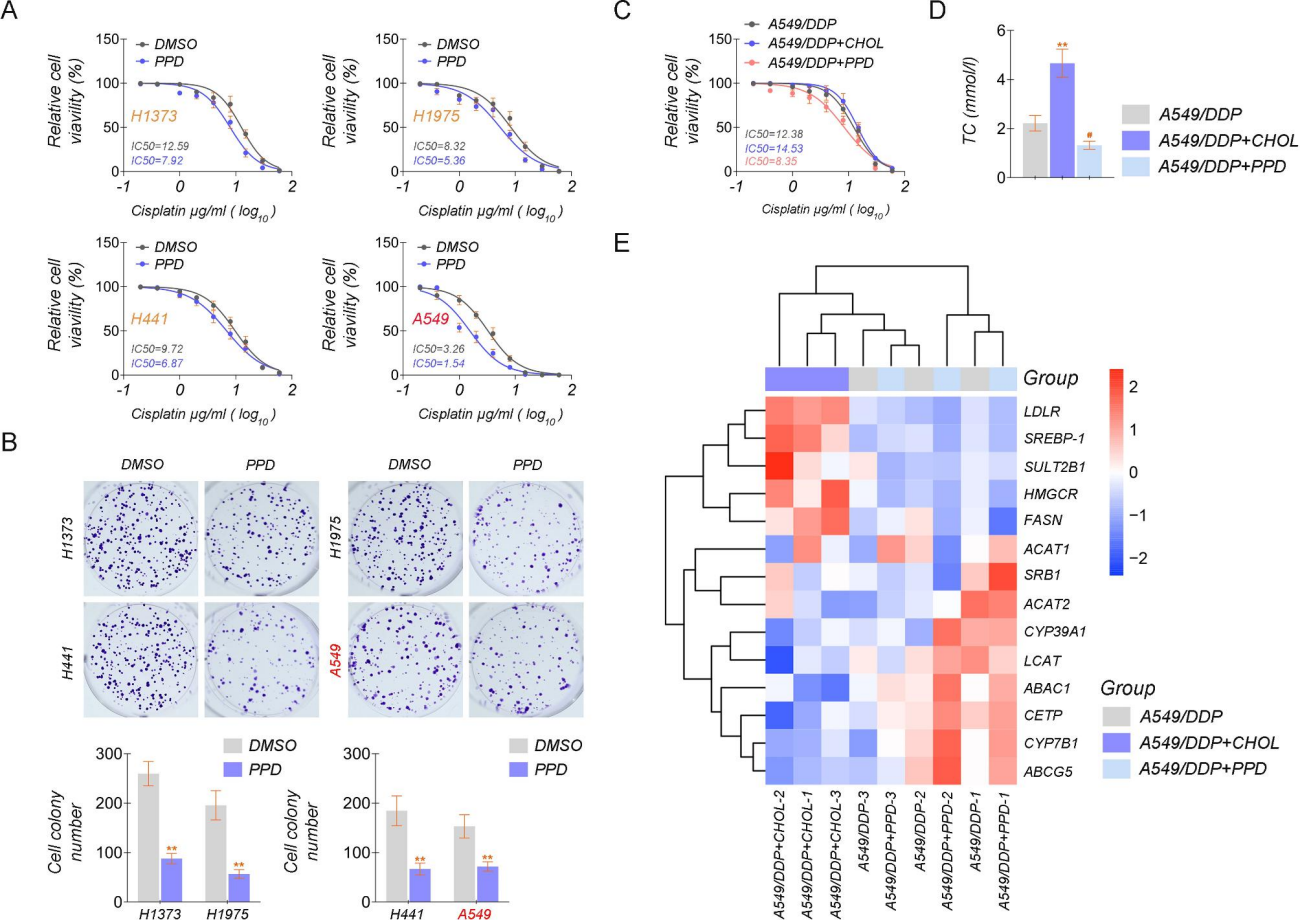
Accumulation of cholesterol reduces sensitivity to cisplatin in lung cancer cell lines **(A)** The IC50 of cisplatin in H1373, H1975, H441, and A549 cell lines treated with PPD or DMSO. **(B)** Colony formation assay in H1373, H1975, H441, and A549 cell lines treated with PPD or DMSO. **(C)** The IC50 of cisplatin in A549/DDP cell lines treated with PPD, CHOL, or DMSO. **(D)** ELISA test kit to detect cholesterol levels (TC) in A549/DDP cell lines treated with PPD, CHOL, or DMSO. **(E)** The cholesterol accumulation-related marker genes were detected. by qRT-PCR in A549/DDP cell lines treated with PPD (Gray), CHOL (Purple), or PPD (Blue). The expression level was displayed from low (blue) to high (red). *PPD* Pseudoprotodioscin, a cholesterol inhibitor, *CHOL* cholesterol. The experiments were repeated three times. For comparisons, a Student’s t-test was performed. ^*^P < 0.05, and ^**^P < 0.01

Considering the correlation between cholesterol accumulation and cisplatin resistance in lung cancer, we selected 4 lung cancer cell lines, H1373, H1975, H441, and A549, treated with DMSO or PPD (Pseudoprotodioscin, a cholesterol inhibitor), to observe whether variations in intracellular cholesterol alter the proliferation of lung cancer cells. The data showed that the decreased cholesterol might inhibit lung cancer cells from death by cisplatin. To varying degrees, the half-maximal inhibitory concentrations (IC50) of lung cancer cells, treated with PPD were lower than that of cells treated with DMSO. And the most significant difference was found in A549 cells (Fig. 1A). Then, the clone formation in 4 lung cancer cell lines was examined, and it further confirmed that the addition of PPD effectively inhibited the proliferation of lung cancer cells (Fig. 1B). To investigate the molecular mechanism of cisplatin resistance linked to cholesterol in lung cancer, we chose the cisplatin-resistant A549/DDP cell line, for further study. We added cholesterol or PPD, observed the IC50 of the A549/DDP cells, and assayed the expression levels of marker genes associated with cholesterol accumulation by qRT-PCR. The IC50 results showed that the addition of PPD reduced the amount of intracellular cholesterol and enhanced the sensitivity of cells to cisplatin (Fig. 1 C-D). We used qRT-PCR to detect a series of genes associated with cholesterol accumulation to determine how cholesterol regulates cisplatin sensitivity in lung cancer cells. The qRT-PCR results showed that the accession of cholesterol increased the expression of sterol regulatory element binding protein 1 (SREBP-1), the essential genes required for sterol biosynthesis (LDLR and HMGCR), and decreased the presentation of the sterol efflux-related gene ABCA1. However, the opposite effect was induced when PPD was added. In this process, the change in HMGCR expression was more dramatic. SREBP-2–mediated mevalonate pathway has been considered as a potential therapeutic approach for lung cancer. And several enzymes, including HMGCR, are regarded as their core downstream targets. Therefore, our later studies will focus on how HMGCR regulates cholesterol accumulation and thus affects lung cancer growth and proliferation and cisplatin resistance. (Figure. 1E).

### Cholesterol biosynthesis induced by HMGCR inhibits cisplatin sensitivity in lung adenocarcinoma cell lines

**Fig. 2:**
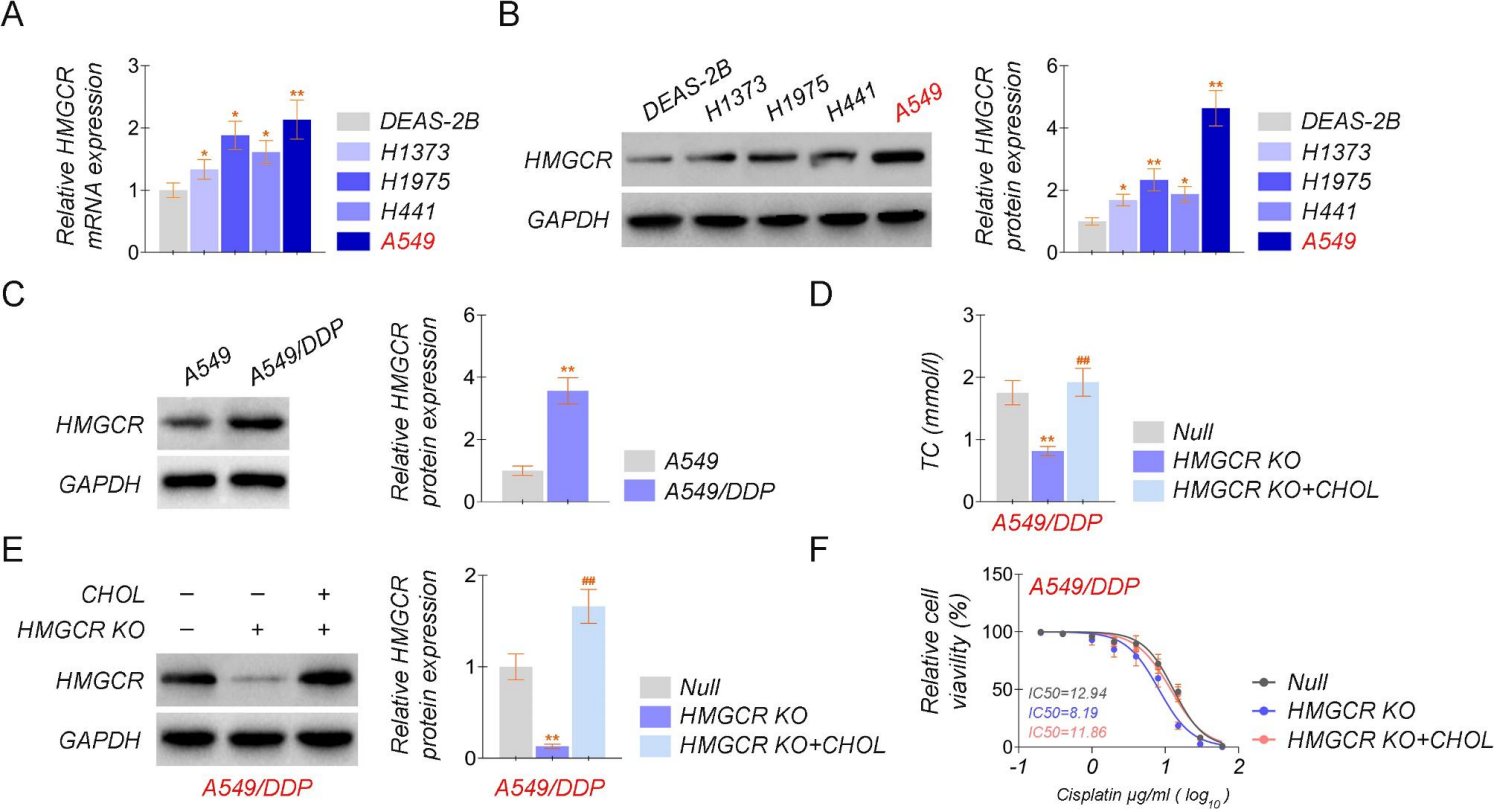
Cholesterol biosynthesis induced by HMGCR inhibits cisplatin sensitivity in lung adenocarcinoma cell lines **(A**) The relative mRNA level of HMGCR in normal cells (BEAS-2B) and lung cancer cell lines (H1373, H1975, H441, and A549). **(B)** The protein level of HMGCR in normal cells (BEAS-2B) and lung cancer cell lines (H1373, H1975, H441, and A549). **(C)** The protein level of HMGCR in A549 and A549/DDP cells. **(D)** ELISA test kit was to detect cholesterol levels with or without cholesterol in the A549/DDP cell lines with knockout HMGCR. **(E)** The protein level of HMGCR with or without cholesterol in the A549/DDP cell lines with knockout HMGCR. (F) The IC50 of cisplatin with or without cholesterol in the A549/DDP cell lines with knockout HMGCR. The experiments were repeated three times. The experiments were repeated three times. For comparisons, a Student’s t-test was performed. ^*^P < 0.05, and ^**^P < 0.01

To better illustrate the effect of HMGCR on drug sensitivity, firstly, we examined the expression of HMGCR in lung epithelial cells and lung cancer cell lines. The expression of HMGCR in lung cancer cell lines (H1373, H1975, H441, A549) was a significant difference in lung cancer cell lines and normal lung tissue cells in the mRNA and protein level of HMGCR (Fig. 2A-B). Then, the expression of HMGCR in A549 and A549/DDP cells was tested, and the protein level of HMGCR in A549/DDP cells was higher than that of A549 cells (Fig. 2C). Afterward, we knocked out HMGCR expression in A549/DDP cells, without or with the addition of cholesterol, and verified the expression level of HMGCR by qRT-PCR and Western blotting assay, respectively, and detected the level of TC by ELISA, as well as the sensitivity of the A549/DDP cells to cisplatin (Fig. 2D-F). We found that exposure to exogenous cholesterol supplementation upregulated HMGCR expression levels and led to cisplatin resistance, while knockout expression of HMGCR could enhance drug sensitivity, respectively (Fig. 2E-F).

### Effects of HMGCR overexpression or knockout on the proliferation of lung cancer cell lines

**Fig. 3:**
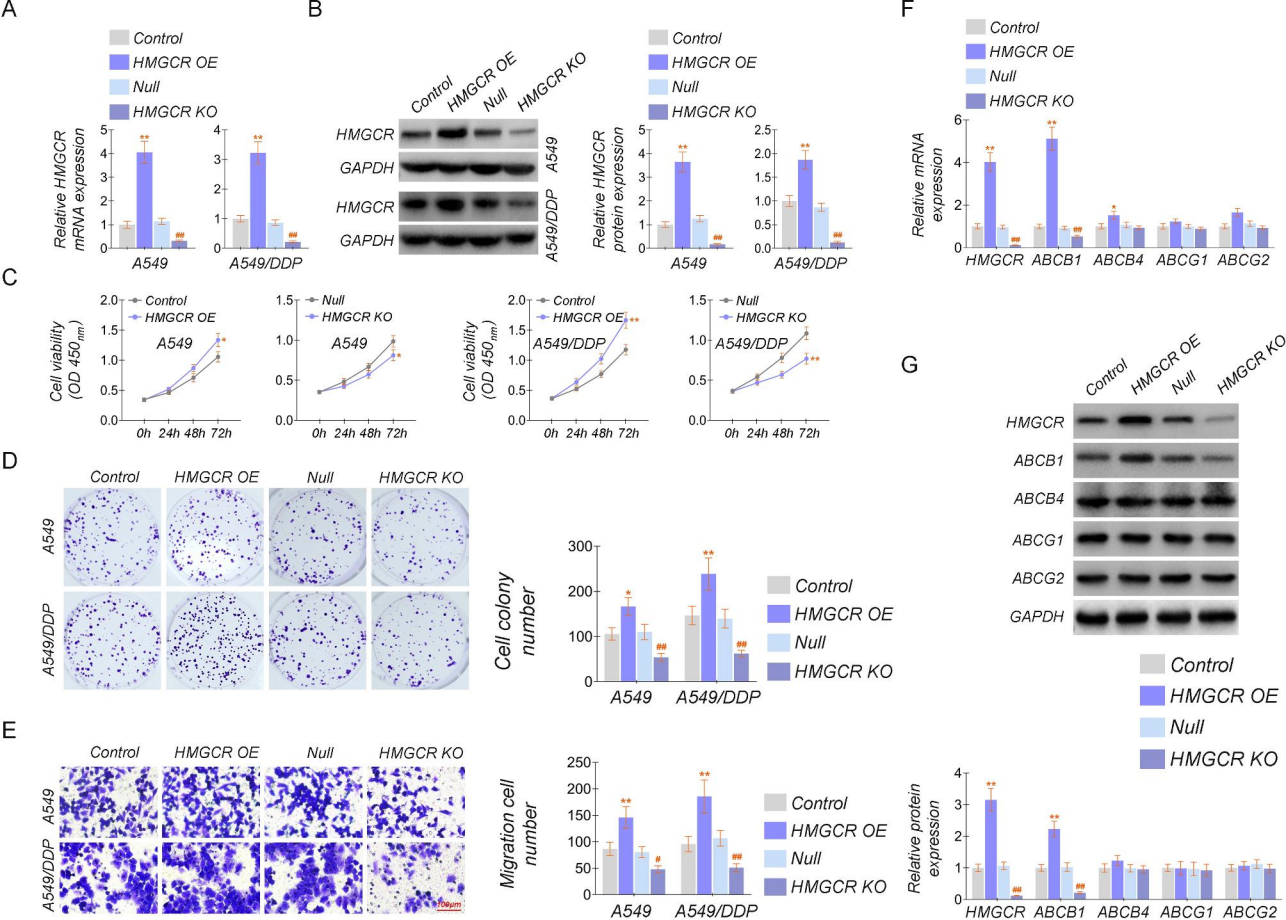
Effects of HMGCR overexpression or knockout on the proliferation of lung cancer cell lines **(A-B)** Detection of relative mRNA and protein levels of HMGCR in A549 and A549/DDP cell lines overexpressing or knocking down HMGCR. **(C)** CCK8 assay was to evaluate the proliferation in the constructed A549 and A549/DDP cell lines overexpressing or knocking out HMGCR. **(D)** Colony formation assay in the constructed A549 and A549/DDP cell lines stable overexpressing or knocking out HMGCR was to test cell proliferation. **(E)** Transwell assay in the constructed A549 and A549/DDP cell lines stable overexpressing or knocking out HMGCR was to evaluate the invasion ability. **(F-G)** The relative mRNA and protein levels of ABCB1, ABCB4, ABCG1, and ABCG2 in the constructed A549 cell lines overexpressing or knocking out HMGCR. The experiments were repeated three times. For comparisons, a Student’s t-test was performed. ^*^P < 0.05, and ^**/##^*P* < 0.01

The above results showed the expression level of HMGCR is not abnormally expressed in lung cancer, but the high expression of HMGCR shows a better prognosis. The function and mechanism of HMGCR in lung cancer need further investigation. The A549 and A549/DDP cell lines stable overexpressed and knocked out HMGCR, and then the cell lines were confirmed by detecting mRNA and protein levels of HMGCR. Both mRNA and protein levels were significantly increased in HMGCR overexpressing cell lines, while in knockout cell lines, both mRNA and protein levels were significantly decreased (Fig. 3A and B). Then, the constructed stable A549 and A549/DDP cell lines were used to study the HMGCR function. The cell proliferation assay was first performed by CCK8 and clone formation experiments, respectively. The results showed that HMGCR overexpression slightly promoted cell proliferation, while HMGCR knockout inhibited somewhat cell proliferation in A549 cell lines. However, HMGCR overexpression significantly contributed to cell proliferation, and HMGCR knockdown extremely depressed cell proliferation in A549/DDP cells (Fig. 3C-D). Transwell assay was employed to examine the effect of HMGCR on cell invasion, and overexpression of HMGCR slightly inhibited cell invasion, while HMGCR knockdown promoted cell invasion in A549 cell lines. The results were similar to cell proliferation. Thefacilitation and repression effects were more pronounced in A549/DDP cells (Fig. 3E).

From the above results, the function of HMGCR overexpression and knockout is outstanding. We speculated that HMGCR restrained drug sensitivity. Therefore, we detected the mRNA and protein levels of drug resistance-related [16], including ABCB1 (P-gp), ABCB4, ABCG1, and ABCG2 in stable overexpressing or knocking out HMGCR A549/DDP cell lines, and found that only ABCB1 mRNA and protein levels changed significantly (Fig. 3F-G). ABCB1 mRNA and protein were dramatically increased in HMGCR-overexpressing cell lines and decreased in HMGCR-knockdown cell lines (Fig. 3F-G). According to the above results, we have initially affirmed the significance of cholesterol homeostasis imbalance triggered by cholesterol hyperaccumulation engaged by HMGCR on cisplatin resistance in lung cancer.

### HMGCR regulates drug sensitivity via the E3 ubiquitin ligase SIAH1

**Fig. 4:**
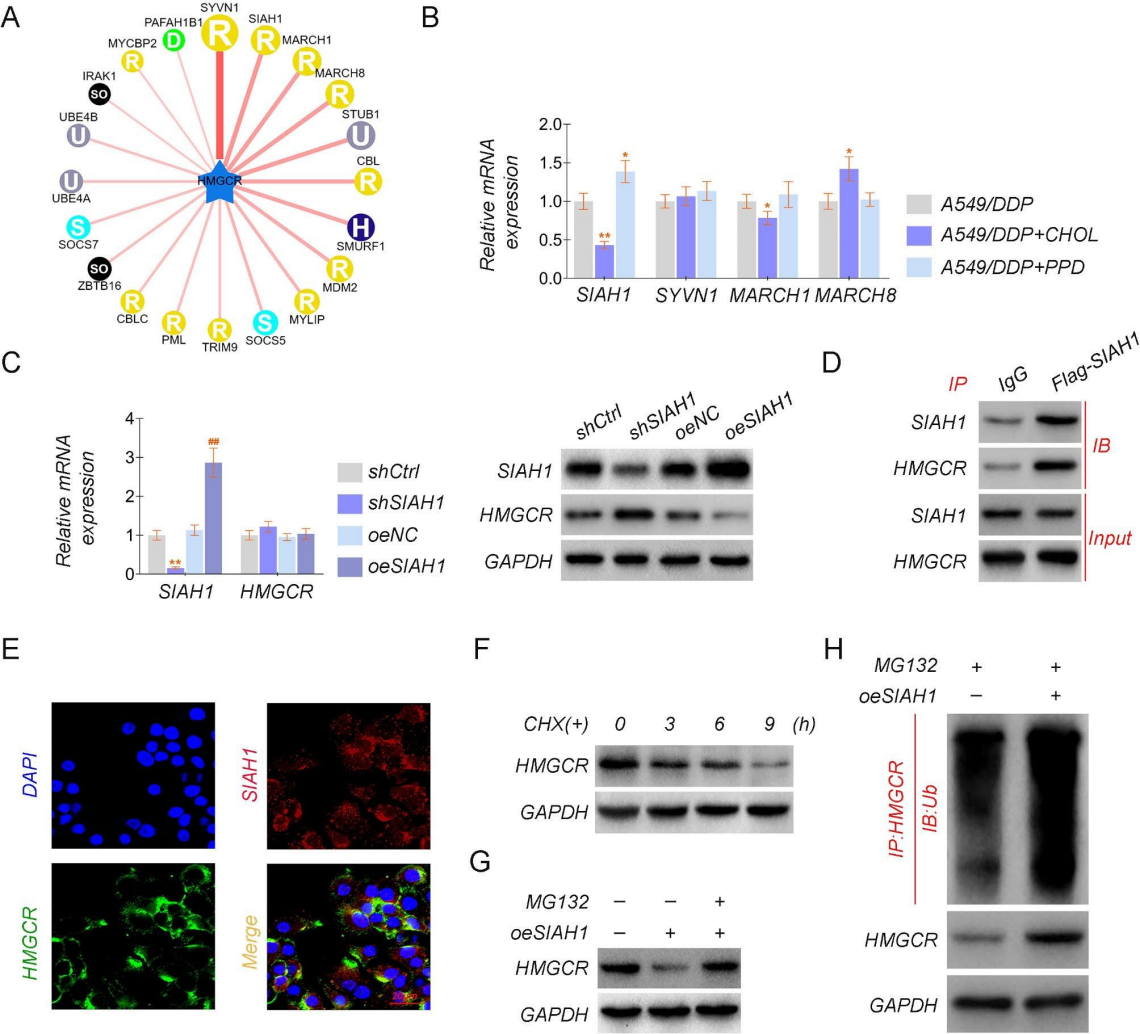
HMGCR regulates drug sensitivity via the E3 ubiquitin ligase SIAH1 **(A)** Ubibrowser predicted the ubiquitinase of HMGCR. **(B)** The relative mRNA and protein levels of SIAH1, SYVN1, MARCH1, and MARCH8 in the A549/DDP cell lines with CHOL or PPD. **(C)** The relative mRNA and protein levels of SIAH1 and HMGCR in the constructed A549/DDP cell lines are stable overexpressing or knocking out SIAH1. **(D)** Flag- SIAH1 was transfected into A549/DDP cells, and then an IP assay revealed the association between SIAH1 and HMGCR. **(E)** Immunofluorescence analysis showed the colocalization of SIAH1 and HMGCR in A549/DDP cells. **(F)** Western blot analysis showed HMGCR protein levels at different time points of actinomycin (CHX, 50 µg/ml) treated A549/DDP cells. **(G)** A549/DDP cells were treated with or without MG132 (10 µM) for 6 h, and then protein was blotted to show HMGCR protein levels. **(H)** The plasmids shown were cotransfected into H293T cells, and the ubiquitination status of HMGCR was determined using an immunoprecipitation assay. The experiments were repeated three times. For comparisons, a Student’s t test was performed. ^*^P < 0.05, and ^**/##^*P* < 0.01

To further elucidate the molecular mechanisms by which HMGCR regulates cisplatin resistance in lung cancer, we found that HMGCR is subject to ubiquitinated structural modifications to stabilize its expression. Ubibrowser (http://ubibrowser.bio-it.cn/) was used for polyubiquitination and its site prediction. We discovered that several ubiquitinating enzymes have regulatory effects on HMGCR, and we selected SIAH1, SYVN1, MARCH 1, and MARCH 8 for the study according to their scores (Fig. 4A). The qRT-PCR data revealed that the addition of cholesterol down-regulated the expression of SIAH1 and MARCH 1 and up-regulated the expression of MARCH 8. In contrast, the addition of PPD up-regulated the expression of SIAH1, but the expression levels of other genes did not change significantly compared with the standard group (Fig. 4B). Next, we constructed A549/DDP cell lines overexpressing and knocking down SIAH1 with the lentiviral vector, and Western blotting was used to assay the expression of SIAH1 and HMGCR. The analysis indicated that the HMGCR protein level was increased following SIAH1 knockdown, while the HMGCR protein level was reduced when SIAH1 was overexpressed. However, the mRNA level remained unchanged, demonstrating that SIAH1 regulates HMGCR at the posttranscriptional level (Fig. 4C). Then, to further elucidate how SIAH1 interacts with HMGCR, we immunoprecipitated Flag-SIAH1, and Western blotting against HMGCR and SIAH1 confirmed that HMGCR interacted with SIAH1, while SIAH1 was discovered after the IP of HMGCR, suggesting that SIAH1 interacted with HMGCR (Fig. 4D). Moreover, confocal laser scanning microscopy demonstrated colocalization of SIAH1 and HMGCR in A549/DDP cells (Fig. 4E). Cycloheximide (CHX) assays revealed that adding CHX decreased the HMGCR protein’s stability (Fig. 4F). Then, we found that MG132, a proteasome inhibitor, restored HMGCR protein expression, which was repressed by SIAH1 overexpression (Fig. 4G). The ubiquitination of HMGCR in cell lysates was revealed by immunoprecipitation with an anti-HMGCR antibody and Western blotting with an anti-UB antibody. The ubiquitination of endogenous HMGCR was promoted by ectopic expression of SIAH1 (Fig. 4H). These findings reveal that SIAH1 physically interacts with HMGCR, preventing HMGCR degradation by regulating its deubiquitination.

### SIAH1 enhances cisplatin sensitivity through HMGCR inhibition of efflux protein activity to inhibit cell proliferation migration invasion

**Fig. 5:**
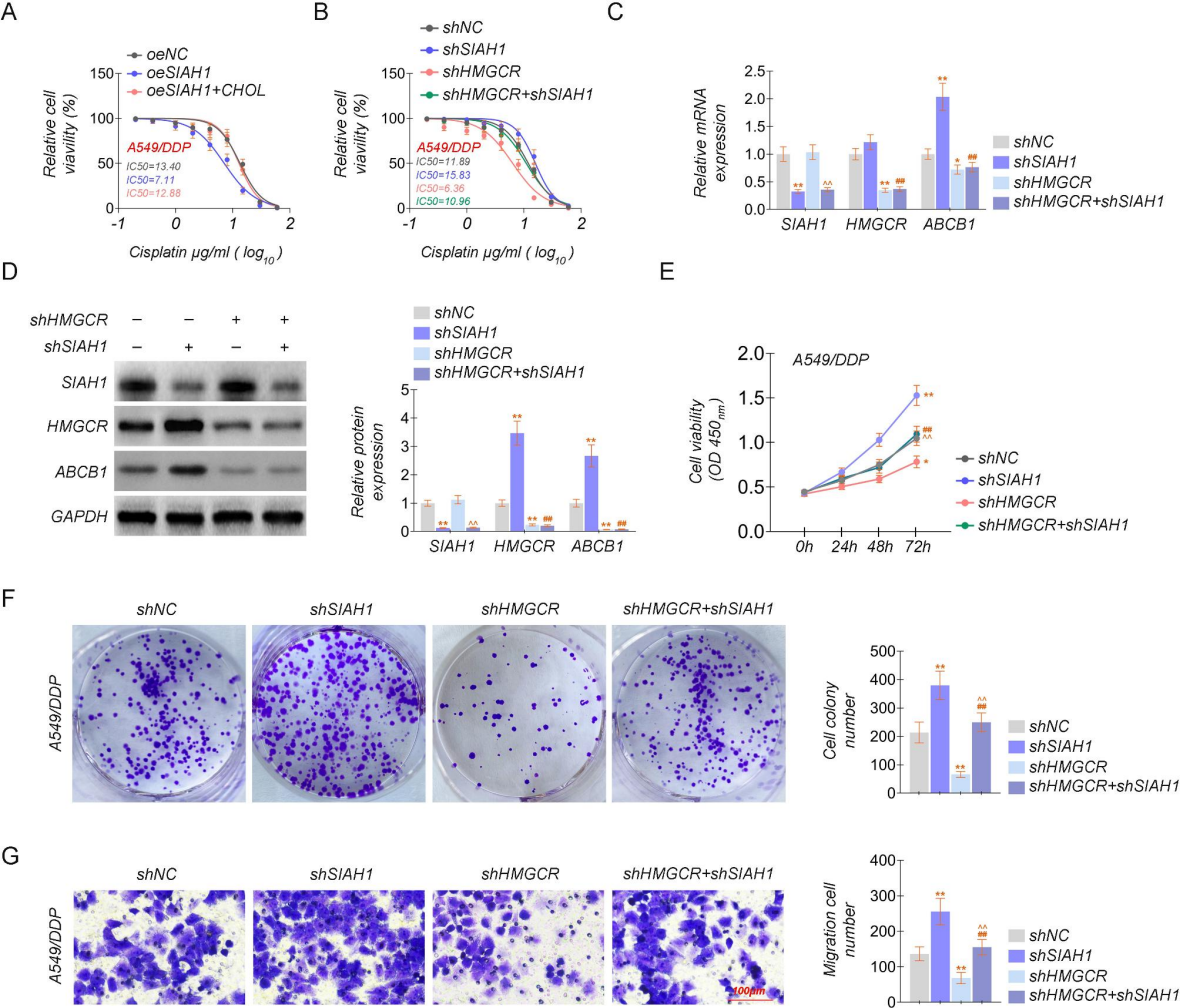
SIAH1 enhances cisplatin sensitivity through HMGCR inhibition of efflux protein activity to inhibit cell proliferation migration invasion **(A)** The IC50 of cisplatin in A549/DDP stable cell lines overexpressing SIAH1 with or without cholesterol. **(B)** The IC50 of cisplatin in the constructed A549/DDP cell lines knocked down SIAH1 and/or HMGCR. **(C-D)** The relative mRNA and protein levels of SIAH1, HMGCR, and ABCB1 in the stable cell lines of A549/DDP with SIAH1 and/or HMGCR knockdown. **(E)** CCK8 assay was to evaluate the proliferation in the constructed A549/DDP cell lines with SIAH1 and/or HMGCR knockdown. **(F)** Colony formation assay in the constructed A549/DDP cell lines knocked down SIAH1 and/or HMGCR. **(G)** Transwell assay was to evaluate the invasion ability in the constructed A549/DDP cell lines with SIAH1 and/or HMGCR knockdown. The experiments were repeated three times. For comparisons, Student’s t test was performed. ^*/#^*P* < 0.05, and ^**/##/^^^*P* < 0.01

The roles of SIAH1 in cisplatin still need to be clarified. We employed stable transfection lines in A549/DDP cells overexpressing and knocking down SIAH1, respectively, to further determine the biological function of SIAH1 in cisplatin chemoresistance. Cisplatin or cholesterol was used to treat A549/DDP cell lines which stable overexpressing or knocking down the expression of HMGCR or SIAH1. Then, the IC50 of cisplatin was calculated. The results show that overexpression of SIAH1 improved the cisplatin sensitivity while knocking down would counteract the effect of clicking down HMGCR, which led to drug resistance. (Fig. 5A-B). Knocking down of HMGCR and SIAH1 was investigated using Western blotting, then detected the mRNA and protein levels of HMGCR, SIAH1, and ABCB1, and found that overexpression of SIAH1 failed to upregulate ABCB1 while HMGCR was knockdown (Fig. 5C-D). Furthermore, with cisplatin treatment, SIAH1 reduced the proliferation, colony-forming ability, and migration capability of cisplatin-treated A549/DDP cells, while HMGCR depletion has the same role, as suggested by the CCK-8, colony formation, and cellular migration assays (Fig. 5E-G). Our data indicated that SIAH1 / HMGCR is a potential key driver of chemoresistance in lung cancer.

### SIAH1 blocks tumor development by enhancing drug sensitivity in vivo through inhibition of cholesterol synthesis by HMGCR

**Fig. 6:**
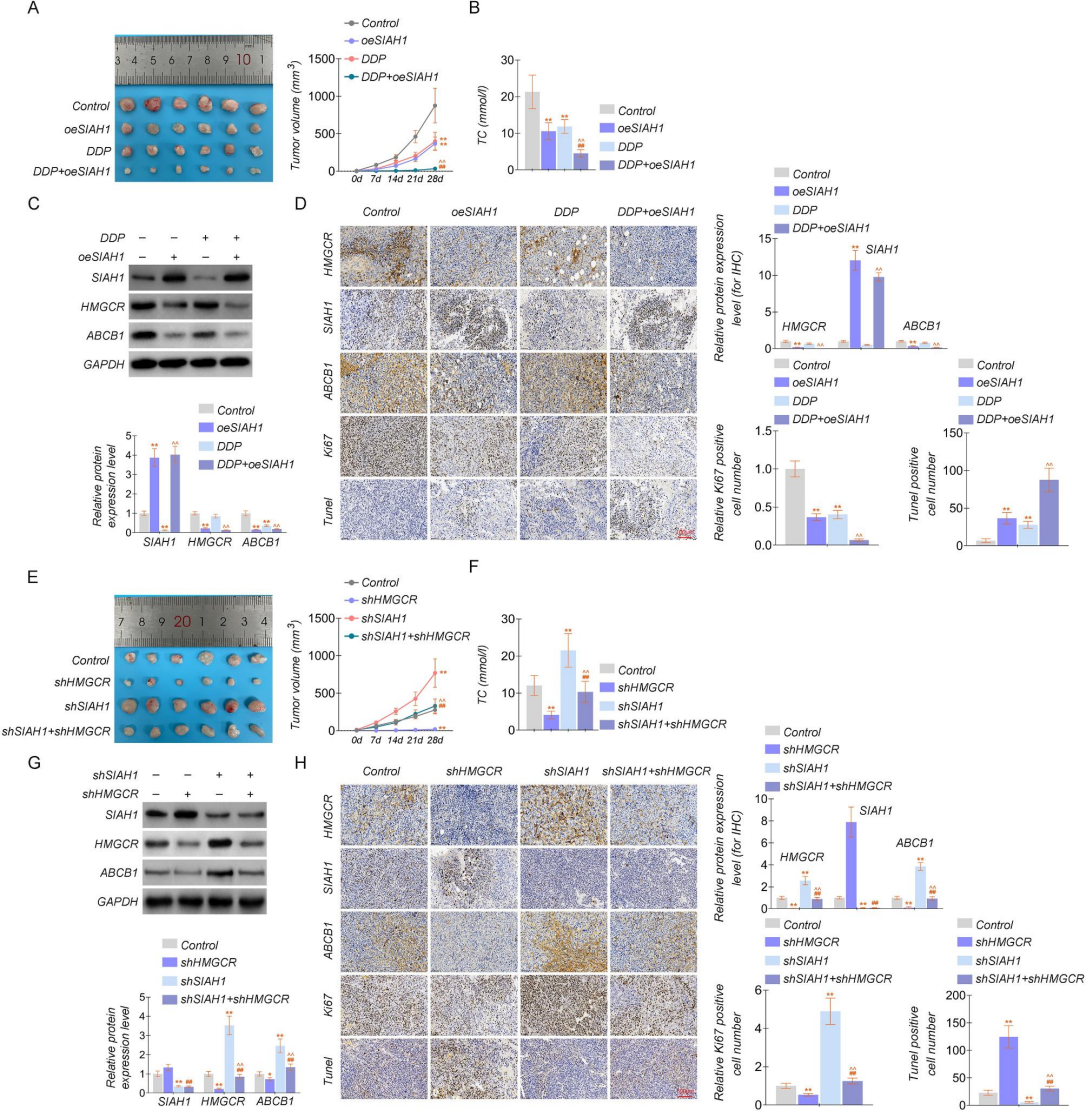
SIAH1 blocks tumor development by enhancing drug sensitivity in vivo through inhibition of cholesterol synthesis by HMGCR **(A)** Tumor volume curve of nude mice under SIAH1 overexpression and/or DDP treatment. **(B) **Detection of TC content in the presence of SIAH1 overexpression and/or DDP treatment. **(C)** The relative protein levels of SIAH1, HMGCR, and ABCB1 were measured under overexpression of SIAH1 and/or DDP treatment conditions. **(D)** IHC detection of SIAH1, HMGCR, and ABCB1 protein expression under overexpression of SIAH1 and/or DDP treatment conditions. And representative images of Ki-67 staining and TUNEL analysis in tumors treated. **(E)** Tumor volume curve of nude mice under SIAH1 or HMGCR depletion. **(F)** Detection of TC content under SIAH1 or HMGCR depletion. **(G)** The relative protein levels of SIAH1, HMGCR, and ABCB1 were measured under SIAH1 or HMGCR depletion. **(H)** IHC detection of SIAH1, HMGCR, and ABCB1 protein expression under SIAH1 or HMGCR depletion. And representative images of Ki-67 staining and TUNEL analysis in tumors treated. The experiments were repeated three times. For comparisons, Student’s t test was performed. ^*^P < 0.05, and ^**/##/^^^*P* < 0.01

To confirm the effect of SIAH1 and HMGCR on sensitive phenotypes in vivo, we established A549/DDP xenografts using nude athymic mice. Firstly, Nude mice were injected subcutaneously with oeSIAH1 and NC cells. After the tumors grew to 100 mm^3^, the mice were treated with or without DDP cisplatin at a dose of 5 mg/kg via intraperitoneal injections every 3 days. At 28 days after the drug injection, the mice were sacrificed, and tumor volumes were determined at indicated days. The results unveiled that the efficacy of cisplatin was significantly improved in the SIAH1 overexpression model (Fig. 6A) and indicated that SIAH1 enhanced cisplatin sensitivity. Meanwhile, SIAH1 could decrease TC content and have a more muscular tumor suppressive effect (Fig. 6A-B). Overexpression of SIAH1 significantly suppressed the expression of HMGCR and ABCB1 protein expression levels, which had a contrary impact on DDP (Fig. 6C). IHC was used to detect the levels of SIAH1, HMGCR, and ABCB1 in xenograft tumors. The results indicated that SIAH1 overexpression promoted cell apoptosis while treated with cisplatin, and the levels of HMGCR and ABCB1 decreased in SIAH1-overexpressing tumors and increased when adding DDP (Fig. 6D). The findings concord with our in vitro results, showing a strong negative correlation between SIAH1 and HMGCR in tumor tissues. Above all, we demonstrated that SIAH1 could regulate the level of ABCB1 through HMGCR and potentiate cisplatin sensitivity. Furthermore, enhanced Ki-67 expression in SIAH1-overexpressing tumors with or without DDP was detected, while the Tunel has the exact reversed situation (Fig. 6D).

Moreover, nude mice were injected subcutaneously with shSIAH1, shHMGCR, and NC cells.

In a vivo mouse model, SIAH1 knockdown promoted tumor proliferation, increased TC content, and the expression levels of HMGCR and ABCB1, consistent with in vitro results (Fig. 6E-G). And HMGCR knockdown inhibited the expression levels of ABCB1 to offset the effect of the knockdown of SIAH1. HMGCR knockdown combination with cisplatin significantly increased TUNEL intensity and reduced Ki-67 intensity, and indicated that HMGCR knockdown promoted cell apoptosis while treated with cisplatin (Fig. 6H). These results proved that SIAH1 affects the ABCB1 level by regulating the protein stability of cholesterol metabolism-related enzymes HMGCR, thereby affecting the cisplatin resistance.

## Discussion

In this study, we found that low HMGCR expression correlated with good prognosis in lung cancer patients and then speculated that HMGCR acts as a tumor promoter. Results showed that HMGCR depletion inhibited cell proliferation in vitro and in vivo. Further studies indicated that SIAH1 inhibited HMGCR expression and enhanced drug sensitivity via downregulating HMGCR to reduce cholesterol synthesis. In vivo experiments showed that SIAH1 overexpression or HMGCR inhibiter in combination with cisplatin inhibits tumor growth significantly.

Substantial experimental data indicates that dysregulation of cholesterol homeostasis correlates with cancer pathobiology. This is not surprising since cholesterol is an essential component of cell membranes and the capacity of cells to increase cholesterol synthesis or accumulation is a precursor to [17, 18]. Cholesterol metabolism is abnormally activated in tumor cells, leading to enhanced proliferation, survival, invasion, metastasis, and adaptation to the tumor [19]. Therapeutic strategies targeting cholesterol synthesis, and lowering plasma cholesterol levels will bring new sight to cancer [20]. In reply, we analyzed cholesterol levels and changes in disease progression in clinical patient information collected and found that high cholesterol was connected with lung cancer progression. In general, this finding is compatible with previous studies, which concluded that cholesterol synthesis is increased in cancer cells compared to untransformed cells, and may promote tumor progression by affecting cell permeability to alter chemotherapy [21]. Relevant clinical trials have been conducted in response to these [22].

HMGCR is a well-known target in cholesterol metabolism, which reduces cholesterol synthesis and thus treats [23]. Recent studies proved that HMCGR inhibitor statins can potentially treat [24, 25]. The mechanism of HMCGR inhibitors in the anti-tumor effect is [26], and the role of HMGCR in cisplatin resistance in lung cancer has not been studied clearly. In our study, HMGCR depletion promoted drug sensitivity, such as cisplatin sensitivity, we suggested that HMGCR inhibitor leads to cisplatin sensitivity via ABCB1 downregulation, as ABCB1 level is associated with cisplatin [27, 28].

We identified that the E3 ubiquitin ligase SIAH1 is responsible for the degradation of HMGCR. SIAH1 has been reported to act as a tumor suppressor during tumorigeneses, such as liver, ovarian, and breast [29, 30]. In addition, SIAH1 has been demonstrated to regulate multidrug resistance 1 (MDR1)/P-glycoprotein-mediated drug resistance in cancer [31, 32]. Previous studies have implicated that SIAH1 degrades HMGCR protein through the ring finger structural domain via the ubiquitin-proteasome pathway, suggesting that HMGCR has a ubiquitinate site. Coherently, our study demonstrated that SIAH1 overexpression reduced HMGCR expression and inhibited the ability of HMGCR to promote proliferation, migration, tumor growth, and DDP resistance in A549/DDP cells, thereby enhancing the chemosensitivity of A549/DDP cells. In addition, double transfection studies confirmed that SIAH1 co-localizes with cytoplasmic HMGCR. Using CO-IP analysis, we determined that the HMGCR is an essential SIAH1-binding protein and that SIAH1 overexpression leads to the degradation of HMGCR via the ubiquitin-proteasome pathway. These values further confirm that SIAH1-mediated ubiquitination of HMGCR may have a significant role in chemical sensitization.

Regarding the connection between HMGCR and P-gp, our study revealed that HMGCR reduces the level of P-gp by regulating cholesterol metabolism, instead of degrading P-gp directly because we found that the mRNA level of P-gp also decreased while HMGCR overexpression. We concluded that HMGCR downregulated P-gp via the subsequent transcriptional regulation. For HMGCR, SIAH1 degraded HMGCR directly, because the protein levels of HMGCR change significantly while the mRNA levels show no changes. Above all, SIAH1 inhibits P-gp levels via HMGCR degradation and cholesterol metabolism dysregulation.

Despite the increasing research on the role of cholesterol in regulating tumor drug resistance mechanisms in recent years, the relationship between p-glycoprotein and cholesterol has not yet been clear, some studies showed that cholesterol modulates p-glycoprotein [33, 34]. Meanwhile, p-glycoprotein mediates cholesterol redistribution in the cell [35]. These studies unveiled that cholesterol levels may affect p-glycoprotein activity without affecting p-glycoprotein protein levels. Another study showed that DAPP (disodium ascorbyl phytostanol phosphate), cholesterol-lowering agents, decreased p-glycoprotein mRNA and protein expression, but NSAS (nanostructured aluminosilicate) has no effect on p-[36]. In our study, SIAH1 decreases cholesterol synthesis via HMGCR degradation reducing p-glycoprotein level. But the relationship between cholesterol and p-glycoprotein needs further elaboration.

In summary, HMGCR affects cholesterol metabolism by regulating of critical enzymes in cholesterol synthesis, thereby depressing drug sensitivity. Mechanistically, SIAH1 ubiquitination regulates HMGCR, blocks cholesterol country accumulation, and inhibits ABCB1 expression, ultimately inducing chemoresistance to cisplatin. Therefore, this study may provide a new potential treatment for cancer, and lung cancer patients with lower HMGCR levels may indicate a better prognosis. And for patients with low HMGCR levels, attempting to control cholesterol intake, or using HMGCR biological inhibitors in combination with cancer therapy may have a better prognosis.

## Conclusions

Our study suggests that HMGCR is a high expression in lung cancer and might be a previously unrecognized new target for lung cancer chemotherapy. HMGCR is regulated by the ubiquitination of SIAH1, which affects ABCB1 activity by disturbing its expression and repressing cholesterol synthesis, thereby enhancing cisplatin sensitivity. Our study delivers new insights and substantial evidence on the clinical characteristics of patients with cisplatin-resistant lung cancer and offers a new treatment strategy.

## Electronic supplementary material

Below is the link to the electronic supplementary material.

Supplementary Material 1

## Data Availability

The authors declare that all data supporting the findings of this study are available within the paper and any raw data can be obtained from the corresponding author upon request.

## Abbreviations

MARCH6: Membrane Associated Ring-CH-Type Finger 6
SQLE: Squalene Epoxidase
HMGCR: HMG-CoA Reductase
P-gp: P-glycoprotein
ABCB1: ATP Binding Cassette Subfamily B Member 1
PVDF: polyvinylidene fluoride
ABCB4: ATP Binding Cassette Subfamily B Member 4
ABCG1: ATP Binding Cassette Subfamily G Member 1
ABCG2: ATP Binding Cassette Subfamily G Member 2
ECL: enhanced chemiluminescence
qRT-PCR: Quantitative Reverse Transcription PCR
CCK-8: Cell Counting Kit-8
IC50: half-maximal inhibitory concentration
IHC: Immunohistochemistry
BSA: Bovine Serum Albumin
PBS: phosphate buffered saline
TCGA: The Cancer Genome Atlas Program
DAPP: disodium ascorbyl phytostanol phosphate
NSAS: nanostructured aluminosilicate
NK: Natural Killer Cells
KD: Knockdown
OE: Overexpression
