# Supplementary material for: SIAH1 ubiquitination-modified HMGCR inhibits lung cancer progression and promotes drug sensitivity through cholesterol synthesis
Source: Cancer Cell Int. 2023 Apr 16;23:71. doi: 10.1186/s12935-023-02914-w (PMC10105949; doi:10.1186/s12935-023-02914-w)
Supplement: Supplementary file 1 — Supplementary Material 1 [file 12935_2023_2914_MOESM1_ESM.docx]

**Table S1 The sequence of primer or shRNA**

| Experiment | Name | Position | Sequence (5’-3’) |
| --- | --- | --- | --- |
| qRT-PCR | SIAH1 | F | TGCTGTTGACTGGGTGAT |
|  |  | R | TGCTGTTGACTGGGTGAT |
|  | HMGCR | F | TTCTTGCCAACTACTTCGTG |
|  |  | R | CATAATCATCTTGACCCTCTG |
|  | ABCB1 | F | TATAATGCGACAGGAGATAGG |
|  |  | R | TTGCCATTGACTGAAAGAAC |
|  | ABCB4 | F | GTGTTCAACTATCCCACCCG |
|  |  | R | CCATCGAGAAGCACTGTCCC |
|  | ABCG1 | F | ACCTTTCCTATTCGGTTCCTG |
|  |  | R | CTTCATGCCCGTCTCCCTGT |
|  | ABCG2 | F | GCTTATTCAGCCAGTTCCAT |
|  |  | R | AGCCGTAAATCCATATCGTG |
|  | GAPDH | F | GACTCATGACCACAGTCCATGC |
|  |  | R | AGAGGCAGGGATGATGTTCTG |
| shRNA | shSIAH1-1 | Top Strand | CCGGCTGATAGGAACACGCAAGCAACTCGAGTTGCTTGCGTGTTCCTATCAGTTTTTG |
|  | shSIAH1-2 | Top Strand | CCGGCAACTTGGCTATGGAGAAACTCGAGTTTCTCCATAGCCAAGTTGTTTTTG |
|  | shHMGCR-1 | Top Strand | CACCGCAGCTTGAAATTATGTGCTGCTTTGCGA  ACAAAGCAGCACATAATTTCAAGCTG |
|  | shHMGCR-2 | Top Strand | CACCGCAGCACTAGCAGATTTGCACGTCTAC  GAATAGACGTGCAAATCTGCTAGTGCTG |
|  | sh-NC | Top Strand | CACCGTTCTCCGAACGTGTCACGTTTCAAG  AGAACGTGACACGTTCGGAGAATTTTTTG |
